# Supplementary material for: Tooth-Cutting-Induced Maxillary Malocclusion Exacerbates Cognitive Deficit in a Mouse Model of Vascular Dementia
Source: Brain Sci. 2023 May 10;13(5):781. doi: 10.3390/brainsci13050781 (PMC10216022; doi:10.3390/brainsci13050781)
Supplement: Supplementary file 1 [file brainsci-13-00781-s001.zip › brainsci-2383410-supplementary.pdf]

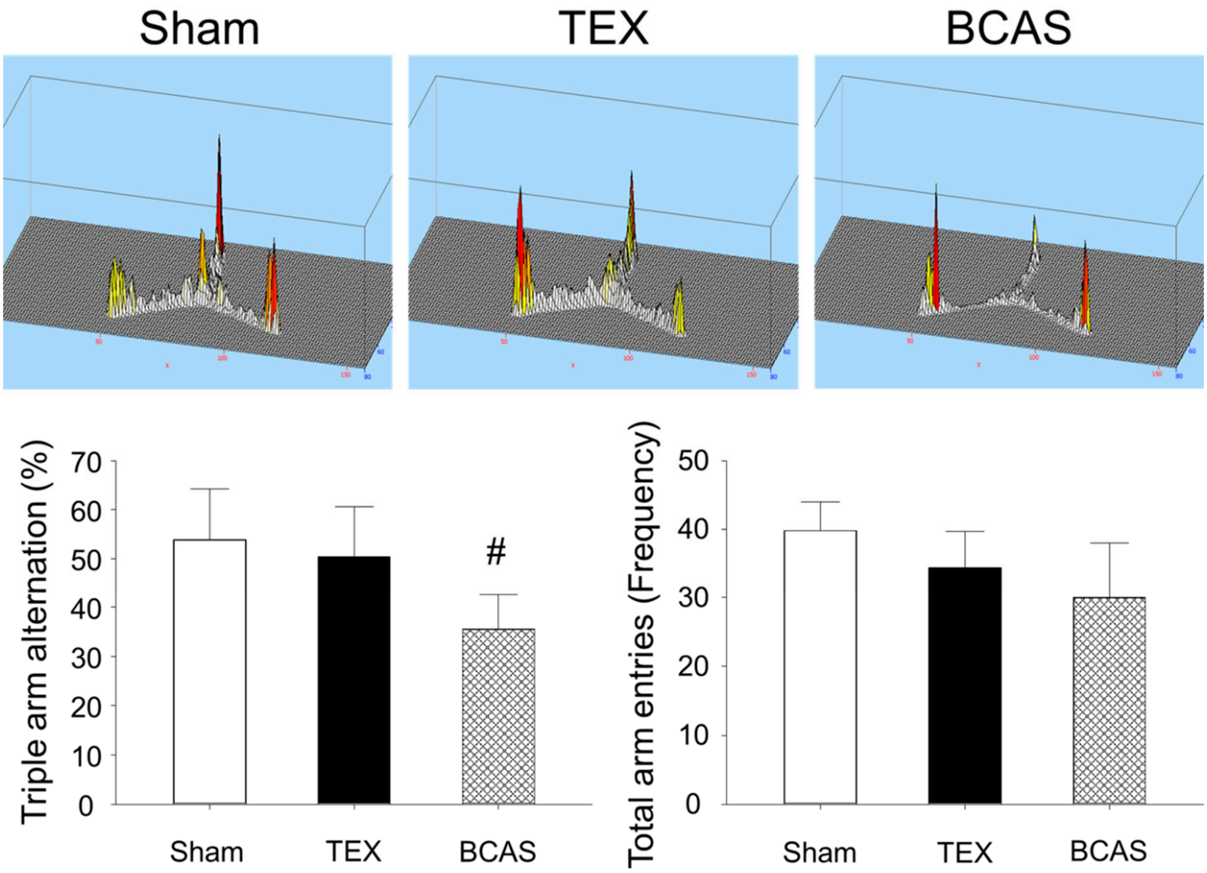

Figure S1. The Y-maze test was used to analyse the changes in the behaviour of mice caused by TEX- or BCAS-induced cognitive function deficit. No significant difference was found between the TEX-only group and the sham group in the triple arm alternation ratio; however, the BCAS group showed a significant (#, $p < 0.05$) difference between the sham group. However, in the DR, no difference was found among the groups.

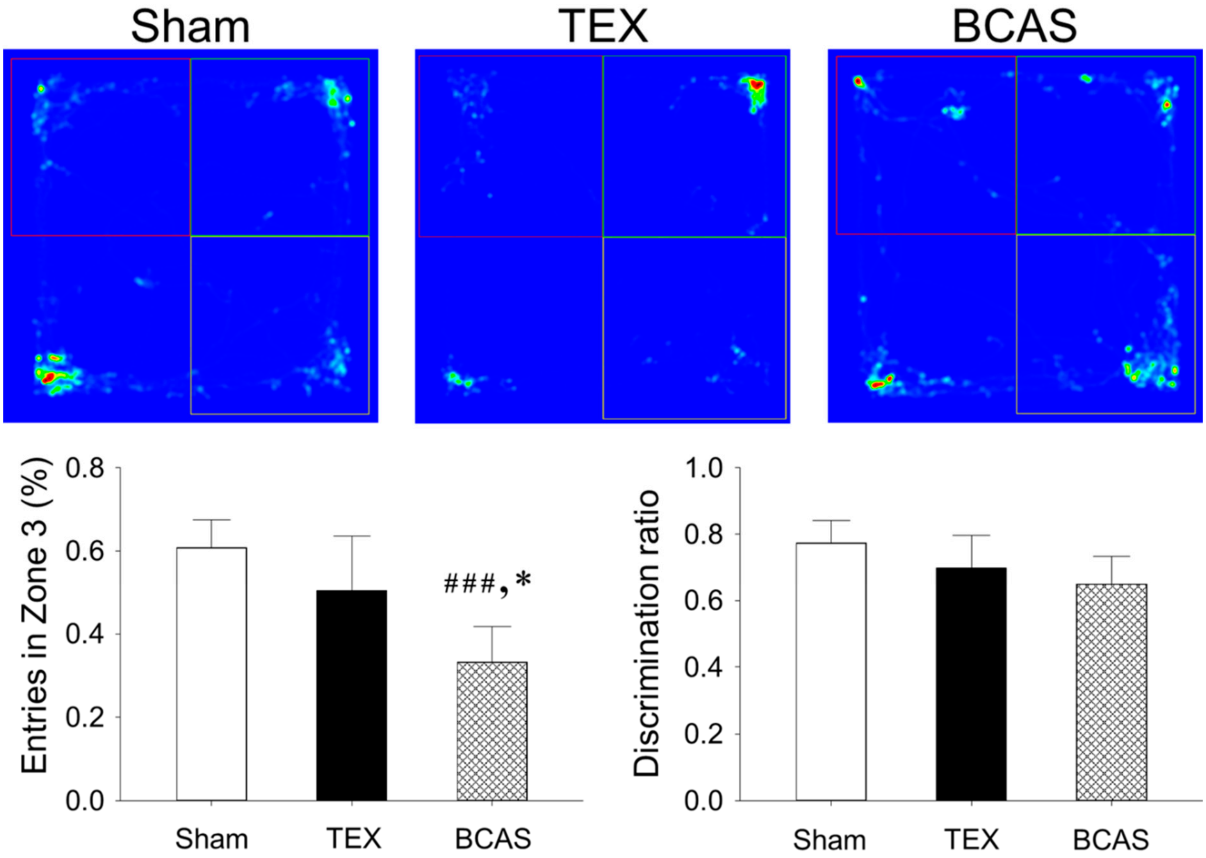

Figure S2. NORT was used to analyse changes in the behaviour of mice caused by TEX- or BCAS-induced cognitive function deficit. When the TEX group was compared with the sham and the BCAS surgery groups, no significant difference was noted, and a difference was found only between the sham and the BCAS groups. In addition, in the case of total arm entries, no difference was found between the sham operation, TEX treatment, and BCAS operation groups ($^{###}$ $p < 0.001$ vs. sham group; * $p < 0.05$ vs. TEX-only group).

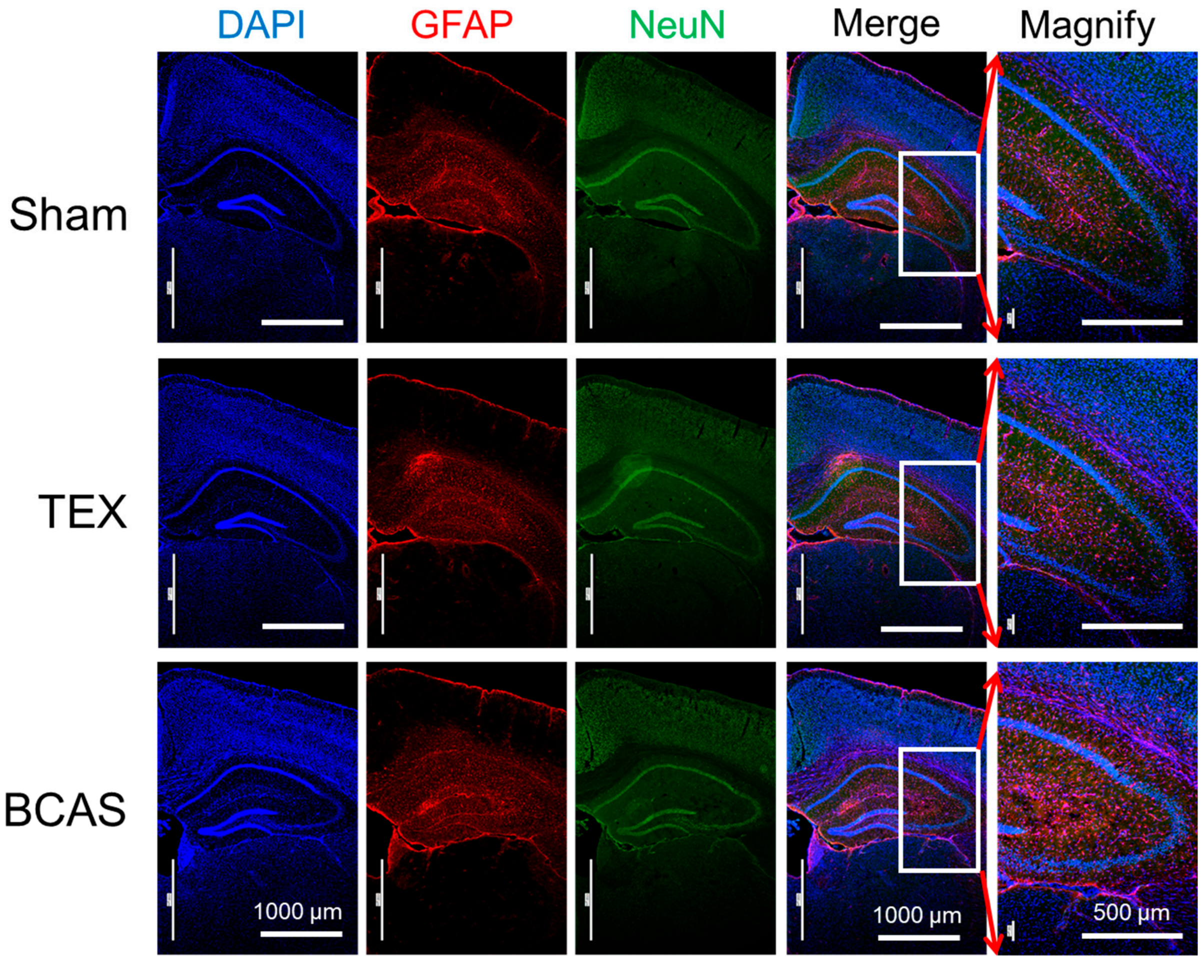

Figure S3. Neuroinflammatory responses in the hippocampal region of TEX- or BCAS-induced mouse brains. Immunofluorescent (IF) staining was performed to confirm the activation of astrocytes. No difference was found in the degree of astrocyte activation between the sham and the TEX-only groups, and astrocyte activation was observed only when BCAS was induced. Each photomicrograph (40× or 100×) represents a region of the hippocampus that was stained using IF.
